# The Immunological Consequences of Pediatric Adenotonsillectomy: Myths, Evidence, and Unsolved Questions

**DOI:** 10.1002/lio2.70556

**Published:** 2026-10-19

**Authors:** Barbara Verro, Gaetano Ottoveggio, Simona Fiumara, Carmelo Saraniti

**Affiliations:** ^1^ Unit of Otorhinolaryngology, Department of Biomedicine, Neuroscience and Advanced Diagnostic (BIND) University of Palermo Palermo Italy; ^2^ Unit of Anesthesia Analgesia Intensive Care and Emergency, Department of Precision Medicine in Medical, Surgical and Critical Care University Hospital Policlinico Paolo Giaccone, Palermo Palermo Italy; ^3^ Speech Therapist Private Practice Palermo Italy

**Keywords:** children, immune system, immunity, tonsillectomy

## Abstract

Tonsillectomy is one of the most common surgical procedures in pediatric populations, indicated primarily for recurrent tonsillitis and upper airway obstruction. Palatine tonsils represent lymphoepithelial structures crucial for local and systemic immune surveillance, with a key role in lymphocyte maturation and the production of immunoglobulins, particularly secretory IgA. This review summarizes the available evidence on the immunological impact of tonsillectomy, including short‐ and long‐term studies. Data show that tonsillar removal can result in transient changes in immunoglobulin and lymphocyte levels, often associated with resolution of a chronic inflammatory state, without compromising the global immune response. Clinical studies also indicate a benefit in reducing recurrent febrile episodes, such as in PFAPA syndrome. Some evidence suggests possible long‐term effects, such as an increased risk of autoimmune or periodontal diseases, especially if the intervention is performed at an early age, although the causality is not yet clear. In conclusion, tonsillectomy performed according to selective clinical criteria generally appears immunologically safe and may provide targeted therapeutic benefits under conditions of local or systemic tonsil dysfunction.

## 1 Introduction

Tonsillectomy is one of the most common surgical procedures in pediatric age, performed primarily to treat recurrent tonsillitis or to improve sleep quality in the cases of obstructive sleep apnea. The palatine tonsils, part of Waldeyer's lymphatic ring, play a crucial role in the immune system, acting as a barrier against inhaled or ingested pathogens. As demonstrated by the ultrastructural study by Jović et al. [1], the tonsillar surface is characterized by deep epithelial crypts rich in immunocompetent cells. This particular architecture facilitates direct contact between external agents and the underlying lymphoid tissue, where there are lymphatic follicles with active germination centers, home to intense proliferation and differentiation of B lymphocytes. Alongside these, a high concentration of T lymphocytes, dendritic cells, and macrophages is observed, evidence of an interaction between humoral and cell‐mediated responses [2]. The dense vascular and lymphatic networks contribute to making the tonsil a real immunological “hub,” able to integrate environmental signals and orchestrate effective mucosal and systemic responses.

Therefore, tonsillectomy raises questions regarding the immunological implications and possible alterations in the long‐term immune response. Previous studies have highlighted that although tonsillectomy can reduce the frequency of acute infections, its consequences on the immune system and susceptibility to chronic diseases remain a matter of debate.

Early work by Arnold [3] suggested that, despite being lymphoid tissues, their removal didn't significantly compromise systemic immunity. In his paper, he reviewed the indications for removing tonsils and adenoids, such as recurrent infections, obstructive symptoms, and complications like peritonsillar abscess. He emphasized that surgical decisions should be based on clinical necessity over theoretical immunological concerns, highlighting the need for careful individual evaluation. Arnold's work reflects the early efforts to weigh the functional significance of tonsils and adenoids against the clear therapeutic benefits of their removal.

Recent guidelines advocate a selective approach, based on well‐defined clinical criteria, such as the Paradise criteria, which identify children most likely to benefit from surgery. These criteria require that the child has had at least seven episodes of tonsillitis in the previous year, or five episodes per year for two consecutive years, or three episodes per year for three consecutive years [4]. Each episode must be documented by a doctor and accompanied by significant clinical signs (e.g., fever ≥ 38.3°C, laterocervical adenopathy, tonsillar exudate or positivity for group A streptococcus). Randomized controlled trials shows that children meeting these thresholds experience significant reduction in frequency and severity of relapses after tonsillectomy, while in cases that do not meet these criteria the benefits are often more modest [5, 6].

Similarly, the 2011 pediatric clinical guidelines of the American Academy of Otolaryngology‐Head and Neck Surgery [7], still considered a reference today, recommend adenotonsillectomy (AT) in children with documented recurrent tonsillar infections according to the same frequency thresholds and clinical documentation. Another major indication is upper airway obstruction, including obstructive sleep apnea or chronic oral breathing due to tonsillar or adenoid hypertrophy. This selective strategy ensures that surgery is clinically justified while minimizing unnecessary removal of lymphoid tissue in children who may not derive substantial benefit.

In light of these considerations, including the dual role of the tonsils as both targets of chronic pathology and active immunological organs, this review aims to critically evaluate the current evidence on the short‐ and long‐term impact of tonsillectomy on pediatric immune function, with the goal of clarifying the balance between therapeutic benefit and potential immunological consequences.

## 2 Search Methodology and Quality Assessment

The review was based on a comprehensive literature search conducted in the PubMed (https://pubmed.ncbi.nlm.nih.gov, accessed on 10 January 2026) and Scopus (https://www.scopus.com, accessed on 10 January 2026), databases using the following search strategy: tonsillectomy OR adenoidectomy OR adenotonsillectomy AND immune system OR immunology.

Inclusion criteria were: studies investigating tonsillectomy, adenoidectomy, and/or AT, retrospective or prospective study designs, and articles published in English or Italian.

The exclusion criteria were: case reports including fewer than five patients, reviews and metanalyses.

A total of 1690 records were identified through database searching. After removing 753 duplicates, two authors (B.V. and C.S.) independently screened the titles and abstracts of the remaining 937 records according to the predefined inclusion and exclusion criteria. Records were excluded based on language (*n* = 236), publication type (case reports, *n* = 105), and lack of relevance to the review topic (*n* = 367). The same reviewers independently assessed the full texts of 229 potentially eligible articles. Any disagreements regarding study eligibility were resolved through discussion and consensus. Fifteen studies met the inclusion criteria through database searching. An additional two eligible studies were identified by screening the reference lists of the selected articles, resulting in a total of 17 studies included in the review (Figure 1) [8].

**FIGURE 1:**
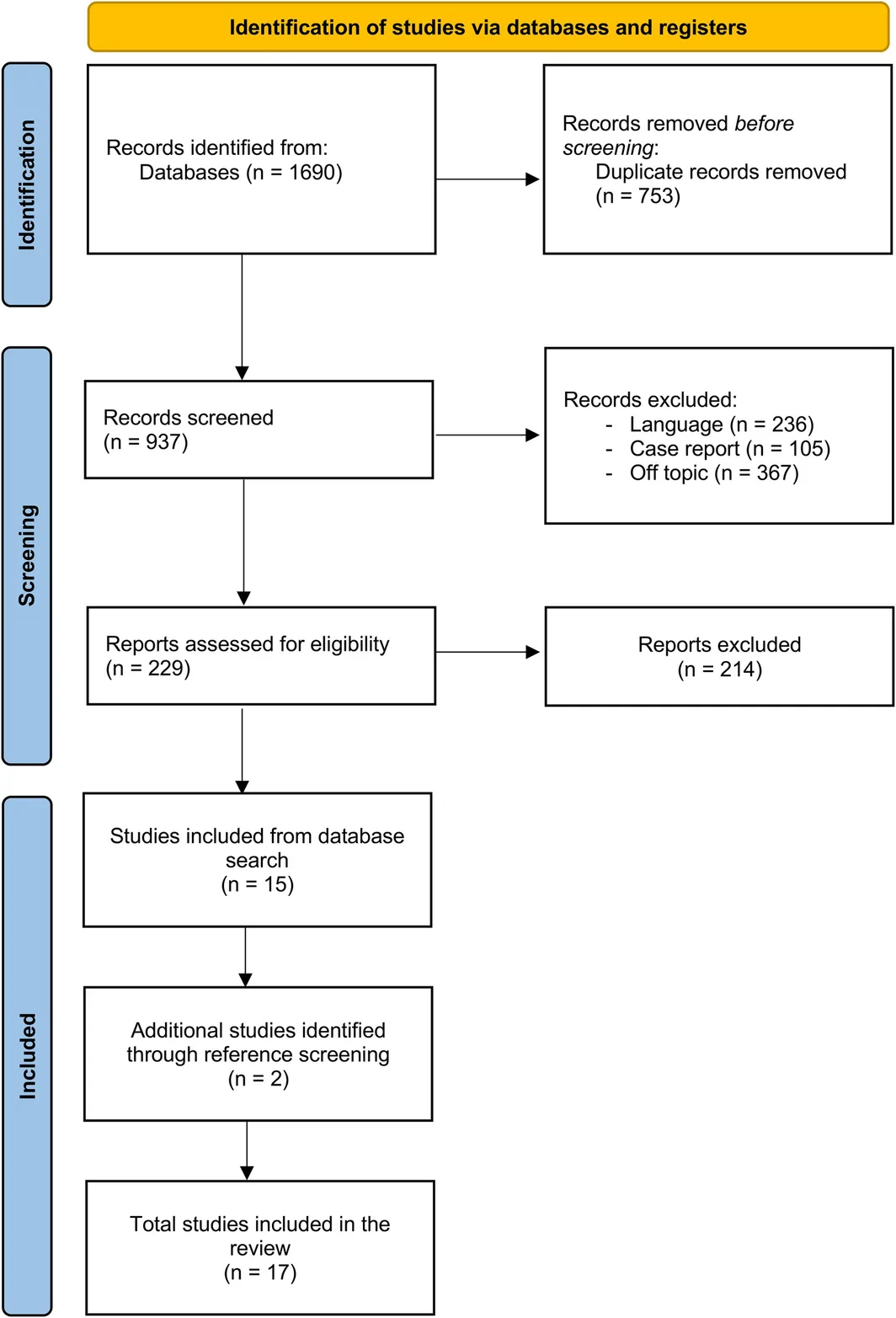
PRISMA 2020 flow diagram of study selection process of the literature.

The methodological quality of the included observational studies was independently assessed by two reviewers (B.V. and G.O.) using the Newcastle–Ottawa Scale (NOS) [9], adapted where appropriate according to the study design. The scale evaluates three domains: selection of participants, comparability of study groups, and assessment of outcomes or exposures, with a maximum score of nine stars. Studies scoring 7–9 stars were considered high quality, those scoring 5–6 stars moderate quality, and those scoring ≤ 4 stars low quality. Any disagreements were resolved through discussion and consensus. The results of the quality assessment are reported in Table 1. Overall, 15 of the 17 included studies were classified as high quality (7–9 stars), whereas two studies were rated as moderate quality (6 stars). No study was classified as low quality.

**TABLE 1:** Methodological quality assessment of the included studies according to the Newcastle–Ottawa Scale.

Study	Design	Selection (max 4)	Comparability (max 2)	Outcome/exposure (max 3)	Total (9)
Böck et al. [10]	Retrospective cohort	****	**	***	9
Childers et al. [11]	Case–control	***	**	***	8
Zielnik‐Jurkiewicz & Jurkiewicz [12]	Comparative study	***	*	***	7
Kaygusuz et al. [13]	Prospective cohort	***	*	***	7
Johansson & Hultcrantz [14]	Cohort	***	**	***	7
Nasrin et al. [15]	Prospective controlled	***	*	**	6
Santos et al. [16]	Prospective cohort	***	*	***	7
Yan et al. [17]	Prospective cohort	***	*	***	7
Marcano‐Acuña et al. [18]	Prospective observational	***	**	***	8
Radman et al. [19]	Retrospective case–control	***	*	**	6
Ma KS et al. [20]	Nationwide cohort	****	**	***	9
Espahbodi et al. [21]	Prospective observational	***	*	***	7
Mostafa et al. [22]	Prospective controlled	***	*	***	7
Murugesan et al. [23]	Family‐controlled cohort	***	**	***	8
Manthiram et al. [24]	Multicenter observational cohort	***	**	***	8
Song et al. [25]	Prospective cohort	***	*	***	7
Ji et al. [26]	Nationwide retrospective cohort	****	**	***	9

## 3 Immune Role of the Tonsils

The palatine tonsils are paired lymphoepithelial structures, located in the lateral pharynx, between the anterior and posterior palatine pillars. They are part of the Waldeyer lymphatic ring, together with the pharyngeal, lingual, and tubal tonsils, constituting an important mucosal immune barrier in the oropharynx. Their surface is characterized by numerous crypts that increase the contact area with inhaled or ingested antigens, allowing efficient immunological surveillance [27].

From a histological point of view, the palatine tonsils are made up of non‐keratinized multilayered pavement epithelium which invaginates to form the tonsillar crypts. Beneath the epithelium there is a rich lymphoid tissue organized into follicles with active germinative centers, responsible for B cells' maturation and the production of immunoglobulins, especially secretory IgA. The tonsils therefore represent a primary site for mucosal immune induction, contributing to defense against respiratory and food pathogens [12].

Consequently, the most recent recommendations suggest a “watchful waiting” approach in children with recurrent tonsillitis who do not meet the Paradise criteria. However, the guidelines also recommend evaluating additional conditions that may alter the surgical decision, including periodic febrile syndromes. In this context, PFAPA syndrome (periodic fever, aphthous stomatitis, pharyngitis and cervical adenitis) is the most common form of pediatric periodic fever [28]. Children with PFAPA have recurrent stereotyped fever episodes with complete remission between attacks, and studies have shown that tonsillectomy often dramatically reduces or eliminates these episodes [6].

Jartti et al. [29] investigated tonsillar immune responses to viral infections, considering age and allergic conditions. Tonsil samples from 143 patients undergoing elective tonsillectomy for tonsillar hypertrophy (34%) or recurrent tonsillitis (30%) were analyzed. By polymerase chain reaction (PCR) for the detection of respiratory viruses and quantitative real‐time PCR (RT‐PCR) for the expression of cytokines and transcription factors, it was possible to characterize the local immune response in relation to the presence of viral infections. At least one virus was detected in 24% of tonsil samples, with significantly higher prevalence in younger children (*p* value < 0.05), suggesting that the tonsils represent an important site of antiviral defense in childhood. In particular, the presence of rhinovirus or parainfluenza virus was associated with increased IFN‐γ and Tbet expression (*p* value < 0.05), indicative of an active Th1 response, while other cytokines such as IL‐37 showed correlations with atopic dermatitis (*p* value = 0.018), and IFN‐α, IL‐13, IL‐28, and Tbet were negatively associated with allergic conditions. Two functional tonsillar profiles emerged: regulatory (IL‐10, IL‐17, TGF‐β, FOXP3, GATA3, RORC2, and T‐bet) and antiviral (IFN‐α, IFN‐β, IL‐28, IL‐29). These findings confirm the tonsils' role in dynamic immune modulation, suggesting that tonsillectomy, by removing a site of immune regulation, could influence the body's ability to respond to infections and allergic stimuli.

As stated above, the pediatric clinical guidelines of the American Academy of Otolaryngology‐Head and Neck Surgery [7] stated that in cases of chronic or recurrent tonsillitis, the normal process of transport and presentation of antigens in the tonsils is altered. Indeed, the M cells of the tonsillar epithelium can be damaged or eliminated, causing direct and uncontrolled entry of antigens. Such a condition leads to a disproportionate expansion of mature B lymphocytes, reducing the generation of early memory B cells destined to become secreting IgA lymphocytes. Tonsillar lymphocytes can then become overloaded and unable to respond effectively to other antigens, impairing the local protective function of the tonsils and the ability to support the secretory immune system of the upper airways. From a clinical point of view, this immunological picture provides a justification for the surgical removal of tonsils affected by chronic or recurrent inflammation, since the compromised tonsillar tissue no longer effectively performs its defensive function.

Finally, the review by Abulikemu et al. [30] highlights that tonsils, through the release of pro‐inflammatory cytokines and the recruitment of activated T lymphocytes, can modulate both local mucosal and systemic immune responses, acting as a bridge between peripheral antigens and general immunity. In conditions of local immune dysfunction, tonsils may become sites of aberrant activation, contributing to recurrent or systemic inflammatory processes, such as pediatric autoinflammatory syndromes (e.g., PFAPA). In this context, tonsillectomy may not only remove inflamed tissue but also modulate local immune circuits, reducing inflammatory mediator production while preserving overall adaptive immunity. This perspective supports the concept of tonsils as dynamic immunoregulatory organs, whose removal can have targeted therapeutic implications in selected cases of immune dysregulation (Figure 2).

**FIGURE 2:**
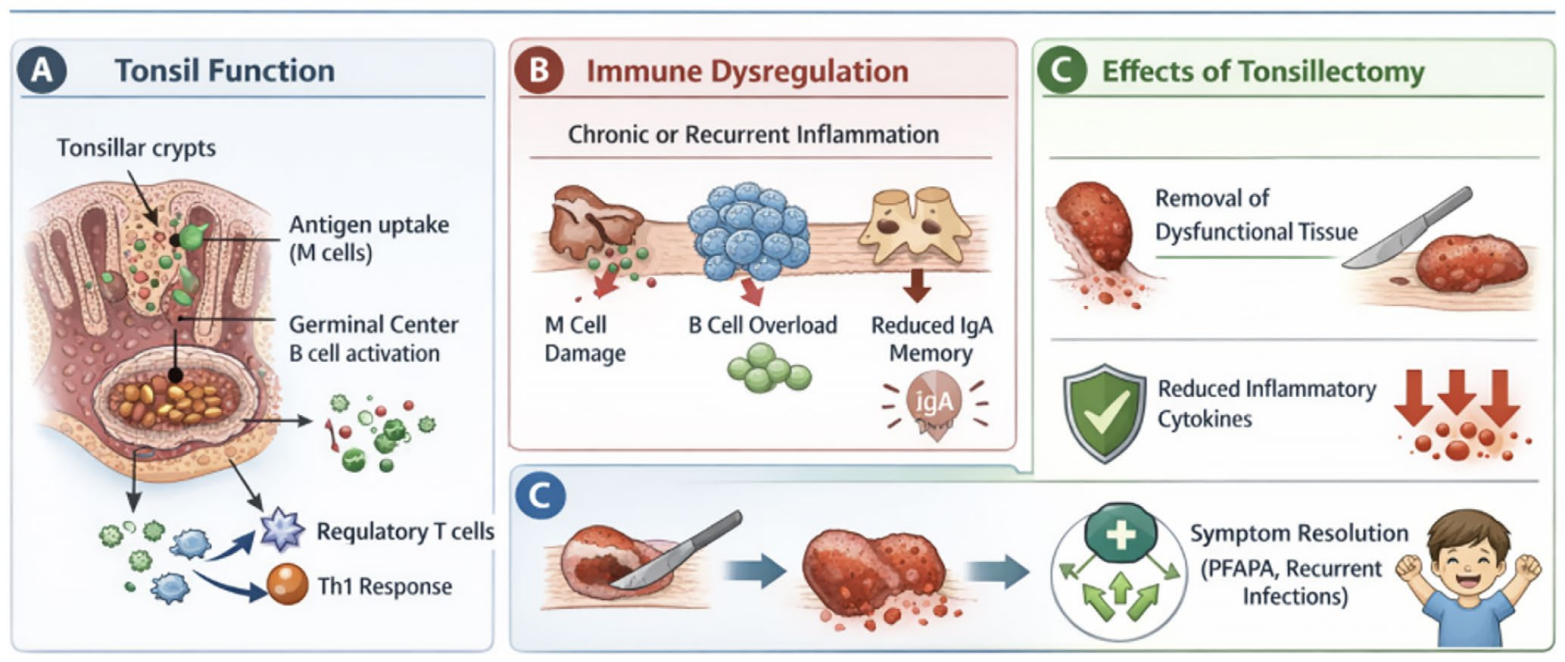
Immunological role of palatine tonsils in childhood: (A) under physiological conditions, tonsils act as inductive sites of mucosal immunity through antigen sampling, germinal center reactions, and IgA production; (B) in chronic inflammation, immune dysregulation leads to persistent activation; (C) tonsillectomy may restore balance by removing a source of aberrant stimulation while preserving systemic immunity.

An important aspect to consider is that the immunological role of the palatine tonsils varies with age. During early childhood, the mucosal immune system is still developing, and the tonsils represent one of the principal inductive sites for antigen recognition, B‐cell maturation, and the generation of secretory IgA. Exposure to environmental microorganisms during the first years of life contributes to the establishment of long‐term mucosal immune memory and immune tolerance. Consequently, removal of lymphoid tissue during this critical developmental period has been hypothesized to exert a greater immunological impact than surgery performed later in childhood, when other components of the mucosal immune system are more fully established and capable of compensating for the loss of tonsillar tissue. However, current evidence remains inconclusive, and available studies suggest that, despite transient alterations in some immunological parameters, clinically significant long‐term impairment of immune function has not been consistently demonstrated [31].

## 4 Short‐Term Evidence

Childers et al. [11] evaluated whether tonsillectomy affects total and specific salivary immunoglobulin levels in children, focusing on oral mucosal immunity, which is an important first line of defense against pathogens. The study compared 25 children who had undergone tonsillectomy from 6 to 14 months with 25 age‐, sex‐, and race‐matched controls, analyzing parotid saliva, total saliva, and serum samples to quantify total IgA, IgA subclasses, and specific antibodies to several antigens relevant to oral and general health. The results showed that there were no significant differences in mean total IgA levels or IgA subclasses in parotid saliva and total saliva between tonsillectomized children and controls, indicating that the ability to produce secretory IgA was not decreased after tonsil removal. However, operated patients had significantly higher levels of specific IgA/IgA1 activity in parotid saliva than controls, suggesting a possible increase in the specific antibody response in some salivary secretions after surgery. No significant differences were observed for specific antibody activity in total saliva or serum IgA and IgG levels between groups. Overall, these data suggest that tonsil removal does not impair secretory IgA production in saliva and may be associated with a modest increase in specific antibody activity in parotid secretions, rather than a reduction in oral mucosal immunity. This finding supports the idea that other components of the mucosal immune system, including residual lymphoid tissues and the salivary glands themselves, may compensate for the loss of tonsillar tissue in generating local antibody responses.

Kaygusuz et al. [13] conducted a prospective study to evaluate the early effects of tonsillectomy on the immune system in children. They enrolled 37 pediatric patients scheduled for tonsillectomy and compared pre‐ and post‐operative (54 months after surgery) immunological parameters. Immunological parameters evaluated included CD3+, CD4+, CD8+, CD19+, CD25+, and CD16++56+ levels for cellular immunity, and IgG, IgA, IgM, C3, and C4 levels for humoral immunity. The results showed: (1) a non‐significant decrease of CD3+, CD8+, and CD19+ levels in the post‐operative period compared to the pre‐operative period (*p* > 0.05); (2) a significant increase in CD4+ level and a significant reduction in CD25+ level in the post‐operative period (*p* < 0.05); (3) statistically significant differences between pre‐ and post‐operative levels of immunoglobulins (IgG, IgA, IgM) and complement components (C3 and C4), which had decreased after tonsillectomy (*p* value < 0.05), but these levels were comparable with those of the control group. In summary, the study suggests that although tonsillectomy may result in slight changes in immunological parameters in the early post‐operative period, these alterations are not clinically significant and a lasting negative impact on children's immune systems is not expected.

The prospective study conducted by Nasrin et al. [15] evaluated the impact of tonsillectomy on humoral immunity in children with tonsillar conditions. A total of 70 patients up to 18 years of age who underwent the surgery and 30 age‐matched control children who had no surgical indication were included. Researchers measured serum IgG, IgM and IgA immunoglobulin levels prior to the surgery and at 1 and 3 months post‐operative follow‐up. Prior to surgery, no significant differences were observed between the operated group and the control group, confirming a similar underlying immune condition. One month after the tonsillectomy, a slight decrease in IgG was detected accompanied by a minimal increase in IgM and IgA, variations which however did not reach statistical significance. At 3 months, levels of all three immunoglobulin classes showed a reduction compared to pre‐operative values and the control group, but only the decrease in IgG was statistically significant. The authors concluded that although tonsillectomy may involve some modification of humoral parameters, the overall impact on humoral immunity is limited and does not significantly impair the body's defensive capacity.

The study by Marcano‐Acuña et al. [18] is a prospective observational study that included 29 children (age 3–9 years) undergoing AT for recurrent tonsillitis or obstructive adenotonsillar hypertrophy, compared with 29 age‐matched healthy controls. Systemic inflammatory markers (high‐sensitivity C‐reactive protein [hs‐CRP], intercellular adhesion molecule‐1 [ICAM‐1], transforming growth factor‐beta [TGF‐β], and a broad panel of Th1 and Th2 cytokines) were assessed preoperatively, at 1 month, and at 12 months post‐surgery. At baseline, intervention candidate patients had significantly higher levels of hs‐CRP, interleukin‐1 (IL‐1), interferon‐γ (IFN‐)γ, tumor necrosis factor‐α (TNF‐α), and ICAM‐1 compared to controls (*p* < 0.05), indicating a state of chronic systemic inflammation. After AT, a significant reduction in hs‐CRP and TNF‐α was observed as early as 1 month (*p* < 0.05), while at 12‐month follow‐up most inflammatory markers were comparable to those of healthy controls, with the sole exception of interleukin‐4 (IL‐4), which remained slightly reduced. The authors interpret these data as indicative of a normalization of the systemic inflammatory state after removal of pathological adenotonsillar tissue. However, serum marker analysis does not allow us to draw definitive conclusions on the impact of AT on immune system maturation or competence, but rather on the resolution of a pre‐existing inflammatory state.

The study by Yan et al. [17, 32] evaluated the short‐ and medium‐term effects of adenoidectomy with or without tonsillectomy on immune function in a prospective cohort of 40 children younger than 3 years operated on for upper airway obstruction (snoring or apnea). Serum immunoglobulin (IgA, IgG, IgM) and complement (C3, C4) levels were measured to assess humoral immunity, while the fraction of various lymphocyte populations (CD3+, CD4+, CD8+, CD4+/CD8+, CD19+, CD56+, CD3+CD4−CD8−, CD3+CD4+CD8+) was analyzed for cellular immune function. Blood samples were collected preoperatively, 1 month later (short‐term effect), and 3 months later (medium‐term effect). At 1 month postoperatively, a significant decrease in IgA levels from baseline was observed (*p* < 0.05), although these values remained within normal ranges for age; no significant changes were found for IgG, IgM, C3, C4, or any of the lymphocyte subpopulations analyzed. At the 3‐month follow‐up (in 30 of the 40 children), no statistically significant differences emerged for any humoral or cellular parameter compared to preoperative values, and no episodes of recurrent respiratory infections or immune‐deficient conditions were recorded during the observation period. The authors conclude that, although adenoidectomy with or without tonsillectomy may result in a temporary reduction in specific antibodies such as IgA in the early postoperative stages, this does not result in significant impairment of overall immune function in very young children. The absence of recurrent infections during follow‐up suggests that the immune system of small patients is able to compensate for the loss of lymphoid tissue, likely due to the plasticity of residual mucosal immunity and the ability of other immune components to maintain an effective protective response.

The study conducted by Espahbodi et al. [21] at the Vanderbilt University Medical Center had as its main objective to evaluate the characteristics and trend of recurrent febrile episodes in children undergoing tonsillectomy. This is a prospective observational survey, conducted over 6 weeks of enrollment, involving a total of 120 pediatric patients, with a median age of 6 years (interquartile range 4–8). The main indications for surgery were obstructive sleep apnea (88%) and recurrent tonsillar infections (33%). Prior to surgery, parents filled out a questionnaire aimed at documenting the frequency and characteristics of febrile episodes in the previous year. The same questionnaires were repeated at 3 and 12 months postoperatively to assess clinical evolution. The authors defined “stereotypical fever” as the presence of recurrent febrile episodes with repetitive and similar clinical characteristics. The analysis of the data showed that 11% of children (13 out of 120) had presented at least six episodes of stereotyped fever in the pre‐operative year. In most of these cases, fever was accompanied by tonsillitis (11 children), cervical adenitis (5) or aphthous stomatitis (3). In the 12 months following tonsillectomy, there was a significant reduction in the frequency of febrile episodes compared to the pre‐operative period (*p* < 0.001), indicating a clinical benefit associated with the surgery. The authors suggest that in pediatric patients who are candidates for tonsillectomy, surgery could be an effective therapeutic option to reduce the symptomatology. At the same time, they emphasize the need for careful preoperative clinical evaluation to identify these patients and optimize postoperative follow‐up. However, the study has some limitations, including the relatively small number of patients, the monocentric nature and the use of parental questionnaires, which can introduce a certain degree of bias. Despite this, the results reinforce the hypothesis of a role of tonsillectomy not only in the control of obstructive and infectious symptoms, but also in the modulation of specific recurrent febrile patterns in pediatric age.

Mostafa et al. [22] evaluated the impact of AT on the immunological and microbiological profile in a pediatric population with chronic adenotonsillar hypertrophy. The study included 35 children undergoing surgery, compared with 35 age‐matched healthy controls, analyzing serological parameters and pharyngeal samples before surgery and at 3 months post‐surgery. In the preoperative period, patients showed elevated pharyngeal bacterial colonization (especially Methicillin‐resistant *Staphylococcus aureus* ), associated with signs of systemic immune activation. After AT, the authors observed a significant reduction in serum IgM, IgG, and IgA levels compared to preoperative values (*p* < 0.01), accompanied by a decrease in IL‐8, a marker of pro‐inflammatory inflammation (*p* < 0.01). These variations were also evident in the comparison with the control group. These findings suggest that removal of pathological adenotonsillar tissue is associated with a modulation of the systemic immune response in the short term, likely related to the resolution of a state of chronic antigenic stimulation rather than actual immune impairment. However, the limited three‐month follow‐up and the absence of functional assessments of cellular and mucosal immunity do not allow definitive conclusions to be drawn on the long‐term immunological impact of the surgery.

Murugesan et al. [23] conducted a family‐controlled cohort study to assess the risk of COVID‐19 infection in children who had undergone AT compared to their non‐operated siblings, using both clinical symptoms and SARS‐CoV‐2 testing and specific antibody measurements. In the AT group, the overall percentage of positivity for COVID‐19 was 80.5%, with 68.9% of symptomatic cases. In the control group, overall positivity was 44%, with only 16% symptomatic positivity. The relative risk of contracting the infection in operated children was 1.8 times higher than in controls, while the risk of symptomatic infections was approximately 4.14 times higher in the AT group. These data, collected in the context of the COVID‐19 pandemic, suggest that pediatric subjects undergoing AT may be more susceptible to SARS‐CoV‐2 infections, and potentially other viral upper airway infections, than their unoperated peers. This finding is consistent with the hypothesis that removal of oropharyngeal lymphoid tissues—key sites of local mucosal immunity—may alter the first line of defense against respiratory pathogens, increasing the frequency or severity of viral infections. However, since this is an observational study with limited sample sizes and family control, it remains difficult to establish direct causality, and the presence of confounding factors (e.g., family exposure, social behavior, presence of comorbidities) requires prudent interpretation of the results.

The study by Al‐Shaikh et al. [33] assessed the level of awareness of the adult population regarding the impact of tonsillectomy on immunity and autoimmune diseases in the city of Abha (Saudi Arabia). A total of 412 participants were included; approximately 25% of participants had undergone tonsillectomy in the past. The results show that over 60% of subjects mistakenly believed that tonsillectomy could permanently reduce immunity, while approximately 45% feared an increased risk of autoimmune diseases. Only 30% reported receiving correct information from medical sources. The authors analyzed some associations with sociodemographic factors and found that higher educational level was significantly related to better knowledge (*p* < 0.05). Although the study does not provide immunological data, it highlights the gap between scientific evidence and public perception, suggesting that a large portion of the adult population holds misconceptions about the immune impact of tonsillectomy. However, the self‐reported nature of the data and the cross‐sectional design limit the conclusions, which may only concern attitudes and knowledge, not actual biological effects.

In their longitudinal investigation, Kirstilä et al. [34] evaluated the effects of tonsillectomy on salivary immunoglobulins, nonimmune antimicrobial agents, and oral microflora in a cohort of 16 adult patients undergoing surgery for non‐malignant conditions. The authors observed: (1) a significant decrease in salivary immunoglobulin A during the first postoperative months with a gradual return to baseline levels after 12 months; (2) immunoglobulin G and immunoglobulin M showed less pronounced variations with no long‐term significant changes; (3) nonimmune antimicrobial factors remained relatively stable indicating that the innate salivary defense system was not substantially affected; (4) the oral microflora exhibited transient alterations with an early increase in potentially pathogenic strains followed by the reestablishment of a balanced microbial ecosystem within 1 year. These findings suggested that the palatine tonsils contribute to the regulation of salivary immunoglobulin A levels and to the maintenance of oral microbial homeostasis, and that tonsillectomy induces temporary but not permanent alterations in mucosal immune responses, particularly affecting adaptive rather than innate defense mechanisms.

Taken together, available studies indicate that tonsillectomy can cause early and generally transient immunological changes, primarily affecting serum immunoglobulins and some parameters of mucosal immunity, without resulting in a clinically relevant immune deficit. The observed variations appear to more often reflect the resolution of a state of chronic inflammation or persistent antigenic stimulation rather than actual impairment of immune function. However, the interpretation of these results must take into account the methodological limitations of available studies, such as small sample sizes, short follow‐up duration, heterogeneity of populations and surgical indications, as well as a predominantly quantitative and serological assessment of the immune response, which does not always reflect its actual functionality. Overall, the absence of consistent evidence of an increase in clinically significant infections in the short and medium term supports the hypothesis of a compensatory capacity of the immune system (Table 2).

**TABLE 2:** Short‐term immunological effects of pediatric adenotonsillectomy.

Author	Study design	Population (age, *n*)	Indication for surgery	Follow‐up (months)	Immune parameters evaluated	Main findings
Childers et al. [11]	Case–control	Children, *n* = 25 AT vs. 25 controls	Recurrent tonsillar disease	6–14	Salivary IgA (total and subclasses), specific IgA, serum IgA/IgG	No reduction in total salivary IgA; increased specific IgA activity in parotid saliva
Kaygusuz et al. [13]	Prospective cohort	Children, *n* = 37	Chronic tonsillitis	5–4	Lymphocyte subsets, IgG, IgA, IgM, C3, C4	Mild changes in T‐cell subsets and immunoglobulins, all within normal range
Nasrin et al. [15]	Prospective controlled	Children ≤ 18 years, *n* = 70 + 30 controls	Tonsillar disease	1 and 3	Serum IgG, IgA, IgM	Mild IgG reduction at 3 months; other changes not significant
Marcano‐Acuña et al. [18]	Prospective observational	Children 3–9 years., *n* = 29 + 29 controls	Recurrent tonsillitis/OSA	1 and 12	hs‐CRP, cytokines, ICAM‐1	Reduction of inflammatory markers post‐surgery
Yan et al. [17]	Prospective cohort	Children < 3 years., *n* = 40	Upper airway obstruction	1 and 3	IgA, IgG, IgM, C3, C4, lymphocyte subsets	Transient IgA reduction at 1 month, normalization at 3 months
Espahbodi et al. [21]	Prospective observational	Children, *n* = 120	OSA/recurrent tonsillitis	3 and 12	Clinical febrile episodes	Significant reduction of recurrent stereotypical fevers
Mostafa et al. [22]	Prospective controlled	Children, *n* = 35 + 35 controls	Chronic adenotonsillar hypertrophy	3	IgG, IgA, IgM, IL‐8, pharyngeal microbiota	Decrease in immunoglobulins and inflammatory markers
Murugesan et al. [23]	Family‐controlled cohort	Children with AT vs. siblings	AT	Pandemic period	SARS‐CoV‐2 infection, antibodies	Higher infection and symptomatic rates in AT group

Abbreviations: AT, adenotonsillectomy; OSA, obstructive sleep apnea.

## 5 Long‐Term Evidence

Böck et al. [10] conducted a retrospective study to examine the long‐term effects of tonsillectomy on the immune system in children. They compared 160 children who underwent tonsillectomy (mean age 6.6 ± 2.1 years) with 302 age‐matched control children who did not undergo tonsillectomy. Immunological parameters evaluated included incidence of upper respiratory tract infections, percentage of CD21+ cells, number of CD4+ cells, presence of DR+ cells and CD4+ DR count, as well as serum immunoglobulin levels (IgA, IgG, IgM). The results showed that the incidence of upper respiratory tract infections was not increased in children undergoing tonsillectomy compared to controls. Mild increases in CD21+ cell percentage, CD4+ cell number, DR+ cells and CD4+ DR count were observed, especially in boys undergoing tonsillectomy, while lymphocyte subpopulations in girls showed no significant differences. IgA levels were lower in children undergoing tonsillectomy, but no significant alterations were observed in the complement system. In summary, the study suggests that although tonsillectomy may result in slight changes in immunological parameters, these alterations are not clinically significant and an increase in the frequency of long‐term immunomodulated diseases is not expected.

Zielnik‐Jurkiewicz and Jurkiewicz [12] conducted a comparative study to examine immunological alterations in children with adenoid and tonsil hypertrophy, comparing them to a healthy control group. The study included 80 patients (aged 3 to 14 years) with adenoid and tonsil hypertrophy, and 40 healthy subjects (aged 3 to 15 years) with no history of recurrent upper respiratory infections. All participants underwent tests to evaluate serum levels of immunoglobulins A, G, and M, the percentage of T lymphocytes (CD3), helper T lymphocytes (CD4), and cytotoxic T lymphocytes (CD8). The study showed that patients with adenoid and tonsil hypertrophy presented significantly higher serum levels of immunoglobulins A, G, and M (*p* value < 0.05) and total T lymphocytes (CD3), helper T lymphocytes (CD4), and cytotoxic T lymphocytes (CD8) (*p* value < 0.01) than the control group. Six months after the AT, all immunological parameters examined returned to the levels observed in the healthy control group (*p* value < 0.01). These results suggest that tonsils and adenoids play an important role in the modulation of humoral and cell‐mediated immunity, and that AT induces transient alterations in these parameters, without compromising long‐term immune function.

Johansson and Hultcrantz [14] conducted a cohort study to examine the clinical consequences of tonsillectomy 20 years after surgery. They involved 18 patients who underwent tonsillectomy in childhood and 54 age‐matched control subjects. A detailed questionnaire was sent to both groups to collect information on upper respiratory tract infections, chronic diseases, antibiotic use, absences from work, and medical visits. The results showed that the frequency and duration of upper respiratory tract infections were similar between the two groups, with an average of approximately 2.5 episodes per year. However, patients undergoing tonsillectomy had a significantly higher prevalence of chronic diseases (asthma, goiter, ulcerative colitis, and arterial hypertension) than controls, with a relative risk of 9.41 (*p* value = 0.024). There were no significant differences in antibiotic use, absences from work, or doctor visits between the two groups. The authors highlighted that, in the tonsillectomy group, the chronic diseases developed later often had an autoimmune component, suggesting a possible link between the intervention and the predisposition to immunological pathologies. They also observed that the children who experienced these conditions did not belong to the younger group but were aged between 5 and 9 years at the time of surgery, which indicates that removing the tonsils at an early age could have a different impact on the immune system compared to operations carried out in the first years of life. This raises the question of whether tonsillectomy can influence the development of the immune response, increasing susceptibility to autoimmune diseases, or whether some individuals already possess a genetic predisposition that leads them to both tonsillar hypertrophy and chronic conditions of immune origin.

The study by Santos et al. [16] evaluated the short‐ and long‐term effects of AT on cellular and humoral immunity in 29 children with adenotonsillar hypertrophy, following subjects preoperatively, at 1–2 months, and at 12–14 months after surgery. Markers measured included absolute helper (TCD4+) and cytotoxic (TCD8+) T lymphocyte counts and serum immunoglobulin IgA, IgM, and IgG levels. In the short‐term period (1–2 months) after AT, a significant increase in CD4+ T count was observed compared to preoperative values (from 1140 ± 563 to 1326 ± 507 cells/mm^3^; mean increase 186 cells/mm^3^, *p* = 0.026), while CD8+ T and immunoglobulins did not vary significantly in this range. In long‐term follow‐up (12–14 months), analyzed in 14 of 29 patients, IgA and IgG levels were significantly lower than preoperative and short‐term values, although they remained within normal ranges for age, while there were no significant changes in CD4+ and CD8+ T counts in this final sample. Overall, the authors conclude that AT does not have significant negative impacts on either cellular or humoral immunity in children, either short‐ or long‐term, with the observed variations falling within expected physiological values for patient age.

The retrospective study by Ji et al. [26] examined the association between tonsillectomy and the onset of autoimmune diseases in 179,875 Swedish individuals—both children and adults—who underwent tonsillectomy between 1997 and 2012. These patients were followed until the diagnosis of autoimmune diseases, allowing a thorough assessment of the risk associated with the procedure. The results showed that 5.357 of these individuals were subsequently diagnosed with autoimmune diseases, with an overall standardized incidence ratio (SIR) of 1.34. Some autoimmune diseases showed a particularly marked increase: polyarteritis nodosa presented the highest risk (SIR 2.97), followed by Sjögren's syndrome (SIR 1.99). Other diseases, including rheumatoid arthritis, multiple sclerosis, celiac disease, and autoimmune hypothyroidism, have shown a more moderate, but still significant, increase in risk. The analysis showed that this association was independent of gender, age at the time of surgery, and surgical indication, suggesting a potentially direct effect of tonsillectomy on immune regulation. The authors propose several biological explanations for these findings. Traditionally, T lymphocyte maturation has been attributed to the thymus, but recent studies have shown that the tonsils also host a T lymphocyte development program, producing thymic‐like cell subgroups [35]. Removing the tonsils would then result in the loss of this source of functional T lymphocytes, with possible long‐term effects on the immune system's ability to maintain tolerance and prevent autoimmune responses. Another possible interpretation concerns the frequent use of antibiotics in patients with recurrent tonsillitis before surgery, as some evidence suggests that prolonged exposure to antibiotics may increase the risk of certain autoimmune diseases [36]. However, the database used in this study did not contain detailed information on antibiotic treatments, and the increased risk was also observed in patients operated for tonsillar hypertrophy or sleep disorders, suggesting that the effect of antibiotics, if any, is likely limited. In conclusion, the study highlights that the incidence of some autoimmune diseases is higher in children and adults undergoing tonsillectomy. The results support the hypothesis that tonsils play an important role in immune regulation and prevention of autoimmune diseases, emphasizing the need to carefully evaluate the indication for intervention and to deepen with further studies the immunological mechanisms underlying this association.

A landmark nationwide population‐based cohort study by Byars et al. [37] further expanded the debate on the long‐term consequences of childhood adenotonsillar surgery. Using Danish national health registries, the authors reported that adenoidectomy and tonsillectomy performed during childhood were associated with an increased long‐term relative risk of several respiratory, allergic, and infectious diseases. These findings generated considerable interest because they contrasted with the majority of immunological studies reporting no clinically significant long‐term impairment of immune function after surgery. However, the study was observational and registry‐based, and therefore cannot establish causality. Moreover, residual confounding and indication bias should be considered, as children undergoing surgery may already differ from the general pediatric population in terms of susceptibility to recurrent infections, airway disease, or underlying immune characteristics. Importantly, Byars et al. evaluated long‐term clinical outcomes rather than direct immunological parameters, making their findings complementary rather than directly comparable to studies assessing immunoglobulin levels, lymphocyte subsets, or cytokine profiles. Consequently, although this study highlights the need for careful patient selection and long‐term follow‐up, it does not conclusively demonstrate that AT causes persistent immune dysfunction.

Song et al. [25] prospectively followed 25 children undergoing AT for approximately 4 years, compared them with 23 healthy controls to assess serum levels of specific immunoglobulin E (sIgE). At the start of the study, 14 of the 25 operated children (56%) were sIgE‐positive, while in the control group 3 of 23 (13%) were sIgE‐negative. At the end of follow‐up, no significant changes in sIgE levels or positivity were observed in operated children, nor were any significant differences observed compared to controls. Clinically, nasal symptom and quality of life scores improved significantly after surgery in sIgE‐positive patients (*p* < 0.05), whereas no significant differences were observed in sIgE‐negative patients (*p* = 1.00). Analysis of cytokines IL‐4, IL‐5, and IL‐10 showed increases in sIgE‐negative patients, but no changes were directly attributable to the intervention (IL‐4 *p* = 0.667, IL‐5 *p* = 0.408, IL‐10 *p* = 0.714). These data suggest that AT does not alter systemic atopy but may influence local immune dysregulation, with improved symptoms in sIgE‐positive children.

Radman et al. [19] conducted a retrospective case–control study to evaluate the long‐term effects of tonsillectomy on immune function in pediatric age. The study included 34 children who underwent tonsillectomy 4 to 6 years prior to analysis and 30 healthy controls of similar age. Subjects had been operated on for common indications such as chronic adenotonsillar hypertrophy or recurrent tonsillitis and had no other known immune pathologies. The immunological panel included serum measurement of immunoglobulins IgA, IgM and IgG and expression of surface markers CD4, CD8, CD10 and CD56. The results showed that the mean levels of IgA (400.2 ± 192.5 vs. 1131.5 ± 222.0 mg/mL), IgM (55.0 ± 25.9 vs. 149.3 ± 67.0 mg/mL), and IgG (43.6 ± 24.6 vs. 210.5 ± 94.4 mg/mL) in the tonsillectomized group were significantly lower than in controls (all *p* ≤ 0.0001). Instead, no significant differences in CD4+, CD8+, and CD56+ levels were observed between the two groups, while the expression of CD10, a B‐cell‐associated marker, was lower in tonsillectomized subjects, but with a non‐statistically significant difference (*p* = 0.108). The authors interpret these data as a possible indication of a reduction in B cells and long‐term antibody production in operated children, since IgA, IgM, and IgG are essentially produced by mature B cells. However, the lack of significant differences in major T lymphocytes (CD4+, CD8+) and natural killer cells (CD56+) suggests that basic cellular immunity remains substantially preserved. It is important to note that, although these results highlight an association between tonsillectomy and lower immunoglobulin levels in a cross‐group comparison, the study does not demonstrate a clinically significant immune deficit or explore actual immune function (e.g., response to infections or vaccinations) beyond serum differences. Furthermore, the significant age difference between cases and controls constitutes a potential confounding factor, as does the absence of functional or longitudinal follow‐up‐up beyond the single observation point years later.

The study by Ma et al. [20] originally addressed the possible association between tonsillectomy and subsequent risk of periodontitis, using data from the Taiwan Longitudinal Health Insurance Dataset collected between 1999 and 2013 in a nationwide cohort analysis. After careful propensity score matching, the researchers compared 648 pediatric subjects who had undergone tonsillectomy with 648 non‐surgical controls, all of whom were free of periodontitis at baseline. During follow‐up, 230 cases (35.5%) in the tonsillectomized group were associated with the development of periodontitis, and the Cox model showed a significantly increased risk compared to controls (adjusted hazard ratio HR = 1.31; 95% CI = 1.08–1.59). This increased risk was particularly pronounced in subjects younger than 12 years of age (HR = 1.58; 95% CI = 1.10–2.27) and was most evident beyond 4 years after surgery (HR = 1.82; 95% CI = 1.29–2.59). Most events classified as tonsillectomy‐associated periodontitis corresponded to aggressive and acute forms (HR = 1.37; 95% CI = 1.10–1.71). These findings suggest that, in this population, tonsil removal in early childhood may be followed by an increased long‐term risk of developing periodontal disease, particularly in younger cohorts and with a prolonged interval after surgery. The mechanisms proposed by the authors are hypothetical and could include alterations in mucosal and systemic immunity following loss of tonsillar lymphoid tissue; however, the study does not provide direct immunological data. Importantly, the observational and retrospective nature of the analysis, as well as the use of administrative encodings for the diagnosis of periodontitis, limit the causal interpretation of these findings, requiring further prospective studies with direct clinical measurements to confirm the association.

In a multicenter observational study, Manthiram et al. [24] analyzed the clinical response to tonsillectomy in 97 children with PFAPA syndrome, followed for a median of 49 months after surgery. Overall, 65% of subjects treated at one center and 35% at another center reported complete resolution of febrile episodes after tonsillectomy; a further 25% experienced a reduction in the severity or frequency of episodes, 23% had remission followed by recurrence, while 4% showed no change in postoperative symptoms. Logistic regression analysis identified some preoperative factors associated with a complete response including resolution of attacks with glucocorticoid therapy, the presence of exudative pharyngitis, and the absence of skin rash or myalgia/arthralgia during pre‐operative episodes. Furthermore, in patients who continued to have attacks after tonsillectomy, 79% reported improvement with prophylaxis with histamine H_2_ receptor antagonists such as cimetidine or famotidine, suggesting possible alterations in immune and inflammatory pathways in refractory cases. PFAPA is an autoinflammatory syndrome characterized by recurrent episodes of fever, oral canker sores, pharyngitis, and cervical adenitis, due to episodic dysregulation of innate immunity [38]. In this context, tonsillectomy may work by removing a key site of local immune activation, where the tonsils appear to contribute to the periodic generation of inflammatory foci. The clinical benefit observed in some patients, as reported by Manthiram et al., suggests that surgery may modulate this tonsillar immune dysregulation, reducing the frequency or severity of episodes in selected PFAPA phenotypes.

In the long term, most observational and cohort studies suggest that tonsillectomy is not associated with a clinically relevant increase in recurrent infections nor with a manifest deficit in cellular immunity, with lymphocyte parameters generally overlapping with those of controls. However, some evidence indicates a persistent reduction in serum immunoglobulin levels, particularly IgA, IgG, and IgM, which, while often remaining within normal age limits, suggest a possible long‐term effect on humoral immunity compartmentalization and B‐cell function. Studies on large population cohorts have also reported an increased risk of some immune‐mediated and inflammatory diseases, including specific autoimmune diseases and oral conditions, while other investigations do not confirm a significant impact on atopy or the frequency of respiratory infections. Interpretation of these findings, however, is limited by the predominantly retrospective nature of the studies, the possible presence of preoperative confounding factors, and the lack of direct functional assessments of the immune response. Overall, the data suggest that tonsillectomy may result in long‐lasting modulation of some aspects of immune regulation, without definitive evidence of clinically relevant impairment of global immunocompetence, necessitating careful evaluation of surgical indications and further long‐term prospective studies (Table 3).

**TABLE 3:** Long‐term immunological and clinical outcomes after pediatric adenotonsillectomy.

Author	Study design	Population (age, *n*)	Follow‐up	Immune parameters evaluated	Main findings
Böck et al. [10]	Retrospective	Children, *n* = 160 + 302 controls	Several years	Infections, lymphocyte subsets, Ig levels	No increase in infections; lower IgA; mild cellular changes
Zielnik‐Jurkiewicz and Jurkiewicz [12]	Comparative	Children, *n* = 80 + 40 controls	6 months post‐AT	IgA, IgG, IgM, T‐cell subsets	Elevated pre‐op immunity normalized post‐surgery
Johansson and Hultcrantz [14]	Cohort	Adults post‐childhood AT, *n* = 18 + 54	~20 years	Infections, chronic diseases	Higher prevalence of autoimmune‐related diseases
Santos et al. [16]	Prospective	Children, *n* = 29	12–14 months	CD4+, CD8+, IgA, IgG, IgM	IgA and IgG lower but within normal ranges
Ji et al. [26]	Nationwide retrospective cohort	*n* = 179,875	Up to 15 years	Autoimmune diseases	Increased risk of several autoimmune conditions
Song et al. [25]	Prospective cohort	Children, *n* = 25 + 23 controls	~4 years	sIgE, cytokines, symptoms	No change in atopy; symptom improvement
Radman et al. [19]	Retrospective case–control	Children, *n* = 34 + 30 controls	4–6 years	Ig levels, lymphocyte markers	Lower IgA, IgG, IgM; preserved cellular immunity
Ma et al. [20]	Nationwide cohort	Children, *n* = 648 + 648 controls	> 4 years	Periodontitis risk	Increased long‐term risk of periodontitis
Manthiram et al. [24]	Multicenter observational	PFAPA children, *n* = 97	Median 49 months	Clinical response	Resolution or improvement in most patients

Abbreviations: AT, adenotonsillectomy; PFAPA, periodic fever, aphthous stomatitis, pharyngitis and cervical adenitis.

## 6 The Possible Role of Tonsillotomy

An additional aspect that deserves consideration is the potential influence of the surgical technique on postoperative immune function. Most studies included in the present review evaluated conventional total (extracapsular) tonsillectomy, whereas partial (intracapsular) tonsillotomy has gained increasing acceptance in pediatric practice because it preserves part of the lymphoid tissue and is associated with reduced postoperative pain and hemorrhagic complications. Histological and immunological findings reported by Reichel et al. [39] demonstrated that the residual tonsillar tissue remains immunologically active, supporting the hypothesis that tissue‐preserving procedures may better preserve local mucosal immune function. However, robust comparative data evaluating the long‐term immunological consequences of intracapsular versus extracapsular surgery are currently lacking. This knowledge gap has prompted the design of prospective randomized trials, such as the TOTO study [40], aimed at comparing the effectiveness and long‐term outcomes of both surgical approaches. Future prospective studies should also incorporate standardized immunological endpoints, in addition to clinical and patient‐reported outcomes, to determine whether tissue preservation translates into meaningful differences in immune function.

## 7 Limitations of the Review

This review has several limitations that should be acknowledged. First, the included studies were characterized by considerable methodological heterogeneity with respect to study design, sample size, patient age, surgical indications, duration of follow‐up, and immunological parameters evaluated. Such heterogeneity limited direct comparison among studies and precluded a quantitative synthesis of the available evidence. Second, most studies were observational and included relatively small patient cohorts, with variable methodological quality. Finally, differences in laboratory methods and outcome measures may have contributed to inconsistencies across studies. Therefore, although the overall evidence suggests that pediatric AT does not result in clinically relevant long‐term impairment of immune function, the findings should be interpreted with caution. Further well‐designed prospective studies with standardized immunological outcomes and long‐term follow‐up are needed to strengthen the current evidence.

## 8 Conclusion

Tonsillectomy is one of the most common surgical procedures in pediatric age, with well‐established indications for recurrent tonsillar infections and upper airway obstruction. The palatine tonsils play a key role in local immune surveillance, with functions integrated into both the humoral and cellular responses. Available evidence suggests that, although tonsillar removal may result in transient changes in immunological parameters, such changes rarely have clinical significance or impair the overall immune response.

Short‐term studies report slight fluctuations in immunoglobulins and lymphocytes, often related to the resolution of a chronic inflammatory state, while long‐term data indicate that the frequency of respiratory infections does not increase and that most immunological parameters are within normal values. Some evidence suggests a potential increased risk of autoimmune diseases or periodontal disease, especially if the intervention is performed in the first years of life, but such associations require further prospective studies to clarify their causality.

In summary, tonsillectomy in children selected according to well‐defined clinical criteria appears immunologically safe, and may have beneficial therapeutic effects in settings of chronic inflammation or local immune dysfunction, such as in PFAPA syndrome. Careful evaluation of surgical indications, balancing risks and benefits, remains essential to optimize clinical and immunological outcomes.

## Funding

The authors have nothing to report.

## Conflicts of Interest

The authors declare no conflicts of interest.

## Data Availability

The data that support the findings of this study are available in PubMed at https://pubmed.ncbi.nlm.nih.gov. These data were derived from the following resources available in the public domain: immune system and tonsillectomy, https://pubmed.ncbi.nlm.nih.gov/?term=immune+system+and+tonsillectomy.
